# Photo-Protective and Anti-Inflammatory Effects of *Antidesma thwaitesianum* Müll. Arg. Fruit Extract against UVB-Induced Keratinocyte Cell Damage

**DOI:** 10.3390/molecules27155034

**Published:** 2022-08-08

**Authors:** Sutthibhon Natewong, Cholticha Niwaspragrit, Piyanee Ratanachamnong, Papavee Samatiwat, Poommaree Namchaiw, Yamaratee Jaisin

**Affiliations:** 1 Department of Pharmacology, Faculty of Medicine, Srinakharinwirot University, Bangkok 10110, Thailand; 2 Expert Center of Innovative Agriculture, Thailand Institute of Scientific and Technological Research (TISTR), Pathum Thani 12120, Thailand; 3 Department of Pharmacology, Faculty of Science, Mahidol University, Bangkok 10400, Thailand; 4 Neuroscience Center for Resarch and Innovation, Learning Institute, King Mongkut’s University of Technology Thonburi, Bangkok 10140, Thailand; 5 Biological Engineering Program, Faculty of Engineering, King Mongkut’s University of Technology Thonburi, Bangkok 10140, Thailand

**Keywords:** keratinocyte, *Antidesma thwaitesianum* Müll. Arg. fruit extract, antioxidant, anti-inflammation, ultraviolet B (UVB)

## Abstract

The main cause of most skin cancers is damage from UVB from sunlight, which penetrate the skin surface and induce inflammation. For this reason, this study aims to identify natural products with photo-protection properties and their mode of action by using the UVB-irradiated HaCaT keratinocyte model. *Antidesma thwaitesianum* fruit extracts at 25, 50, and 100 µg/mL recovered cell viability following UVB exposure in a dose-dependent manner. Cell survival was associated with the reduction in intracellular ROS and NO. In addition, we showed that the pre-treatment with the fruit extract lowered the phosphorylation level of two MAPK-signaling pathways: p38 MAPKs and JNKs. The resulting lower MAPK activation decreased their downstream pro-inflammatory cascade through COX-2 expression and subsequently reduced the PGE_2_ proinflammatory mediator level. The photoprotective effects of the fruit extract were correlated with the presence of polyphenolic compounds, including cyanidin, ferulic acid, caffeic acid, vanillic acid, and protocatechuic acid, which have been previously described as antioxidant and anti-inflammation. Together, we demonstrated that the pre-treatment with the fruit extract had photo-protection by inhibiting oxidative stress and subsequently lowered stress-induced MAPK responses. Therefore, this fresh fruit is worthy of investigation to be utilized as a skincare ingredient for preventing UVB-induced skin damage.

## 1. Introduction

The epidermis is the first layer of defense against invasive pathogens and protects us from external damage, such as ultraviolet (UV) irradiation [1]. Among the ultraviolet radiation, UVB radiation (280–320 nm) from sunlight is a major cause of skin inflammation or “sunburn” [2]. Several previous studies indicated that UVB-irradiated fibroblast and keratinocyte cells revealed an elevation of reactive oxygen species (ROS) in all epidermal cell layers [3,4,5], which contributes to cellular oxidative damage and high damage of lipids, proteins, and nucleic acids, consequently leading to skin inflammation and death [6]. Keratinocytes are the predominant cells residing in the epidermis. They are responsible for crucial functions, such as skin immune response [7]. Upon sunlight exposure, UVB directly penetrates through the epidermal layer and damages human epidermal cells. Keratinocytes are one of the targets that quickly respond to UVB radiation. They generate intracellular ROS. Overexposure to UVB results in uncontrolled inflammation [8]. In addition, UVB penetration may result in DNA damage and chronic skin inflammation, which leads to skin cancer development [9,10].

Following keratinocyte exposure to UVB radiation, a variety of ROS, including superoxide anion, hydrogen peroxide, and hydroxyl radicals, were immediately elevated within 1 h [11]. This caused DNA deterioration, DNA damage, and inflammation [6]. Two MAPK signaling pathways, p38 and JNK, are crucially responsible for keratinocytes upon UVB-induced inflammation [12]. Previous studies demonstrated that UVB radiation triggered the activation of p38 and JNK signaling, which increased the phosphorylation and stabilization of p53 [3,13]. Consequently, it upregulated the inflammatory mediator cyclooxygenase-2 (COX-2) expression [12,14], which in turn elevated the synthesis of its metabolite product, prostaglandin E_2_ (PGE_2_) [15]. Therefore, the attenuation of these inflammatory signaling may be a promising effective strategy to prevent and delay chronic skin inflammation and eventually tumorigenesis.

Nowadays, doctors typically prescribe nonsteroidal anti-inflammatory drugs (NSAIDs), especially celecoxib, to treat inflamed skin. Celecoxib inhibits COX-2 enzyme, which is highly associated with skin cancer [16]. Thus, it is used to treat skin inflamed from sunburn. In addition, celecoxib also possesses antioxidant properties. Therefore, it has been developed for transdermal delivery, such as in topical form, for cutaneous disease treatments [17,18]. In addition to conventional drugs, many researchers, including our research group, are interested in studying the photoprotective effects of natural products. Previous studies showed that anthocyanins, an antioxidant from apple, blackberry, elderberry, peach, pear, cherry, and gooseberry, can be used to treat inflammation and oxidative stress from UVB exposure [12,19]. Over the past few years, natural extracts have gathered interest as herbal remedies to help to prevent photoaging and UV-induced skin inflammation. In this study, we found high anthocyanin contents in a tropical fruit, *Antidesma thwaitesianum* [20]. Thus, this study presents an alternative source of natural products that can be used for UVB-induced skin inflammation treatment [21].

*Antidesma thwaitesianum* (*A. thwaitesianum*) is a tropical plant in the Euphorbiceae family. This plant is widely cultivated in Northeast Thailand [22]. Its ripe red fruits are edible and taste sweet and sour. Thus, it has been added as an ingredient in jam, wine, and juice. Significant amounts of bioactive compounds of polyphenols and flavonoids, such as anthocyanin, catechin, gallic acid, quercetin, rutin, terpene, alkaloids, luteolin, tannin, sterols, and saponins, have been found in this fruit. The beneficial effects of the bioactive compounds contained in these plant extracts have been revealed in previous studies, including antioxidant, anti-inflammation, and hypoglycemic effects [23]. Due to its polyphenolic contents, the hexane extract from seed and bark exhibited anti-inflammation following TPA treatment on MCF10A human breast epithelial cells by inhibiting the cytoplasmic IκBα degradation [24]. In addition, these extracts also prevented hydrogen peroxide-induced apoptosis by suppressing pro-apoptotic BAX [24]. It has been shown that extracts from plants in the *Antidesma* genus exhibit anti-inflammation effects both in vivo and in vitro [25,26]. However, there exists no information regarding the photo-protective effects of *A. thwaitesianum* fruit extract to date. Thus, in this study, we aim to identify the anti-apoptosis and anti-inflammatory effects of *A. thwaitesianum* fruit extracts by using UVB-irradiated HaCaT keratinocytes. This finding can help to elucidate the scientific mechanism of protection and suggest the use of this fruit extract in skin care products.

## 2. Results

### 2.1. In Vitro Antioxidant and Total Phenolic and Flavonoid Contents

**Figure 1:**
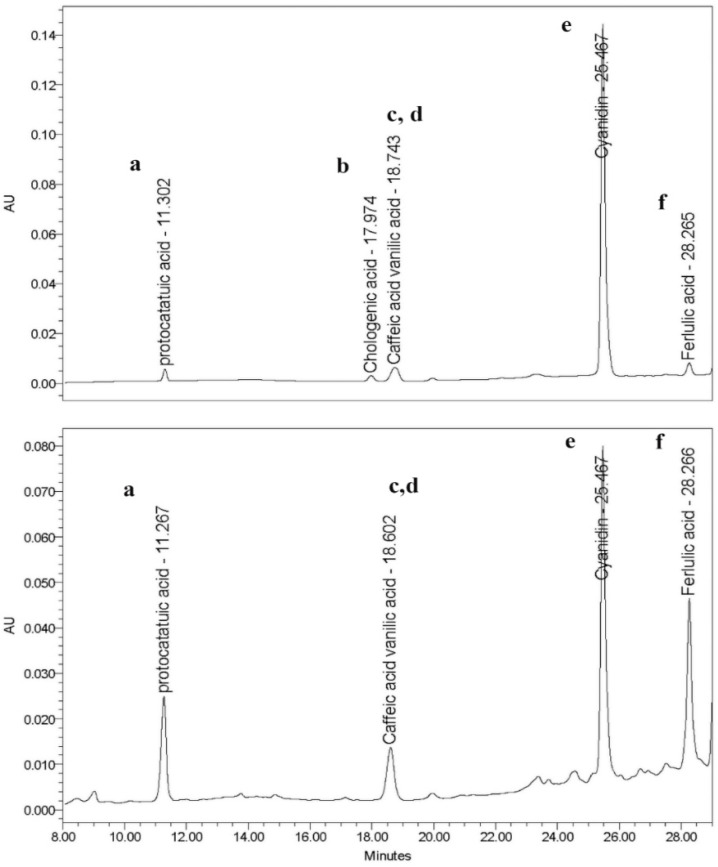
Chromatograms (HPLC/UV) of the fruit extract of *A. thwaitesianum*. The upper figure shows the control compounds, whereas the lower figure shows the active compounds present in the extract. The retention time of the flavonoids was shown as follows: (a) protocatechuic acid, (b) chlorogenic acid, (c) caffeic acid, (d) vanillic acid, (e) cyanidin, and (f) ferulic acid. Compared with the standard compound, the *A. thwaitesianum* fruit extract found five active substances compared to the standard compounds.

Polyphenolic compounds are a chemical structure containing hydroxyl groups attached to an aromatic ring, whereas flavonoid compounds contain two aromatic benzene rings connected with a heterocyclic pyran ring as the linker [27]. Based on their chemical structures, polyphenolic and flavonoid compounds are capable of scavenging free radicals through the electron and proton transfer on hydroxyl groups and generating stable radical intermediates. Thus, it exerts a powerful antioxidant ability [28]. As shown in Table 1, the extract exhibited a total phenolic content equal to 29.115 ± 0.528 mg gallic acid equivalent/g crude extract and flavonoid contents of 1.237 ± 0.104 mg quercetin equivalent/g crude extract. In addition, we showed the DPPH scavenging property of this extract with IC_50_ of 10.94 mg/mL compared with the antioxidant control; Trolox had IC_50_ of 0.17 mg/mL. Along with the scavenging capability of DPPH radical, we further investigated the chemical constituents of this fruit extract using HPLC analysis. We found that the highest peak (most predominant) was cyanidin with the retention time of 25.567 min, while the second peak was ferulic acid, with the retention time of 28.266 min. In addition, we also identified three more peaks, which were identified as caffeic acid, vanillic acid, and protocatechuic acid (Figure 1). The contents of the active compounds present in the *A. thwaitesianum* fruit extract were quantified from the equation of the standard curve, as shown in Table 2. These results indicate that the fruit extract was enriched with polyphenols and flavonoids. In addition, it also displayed free radical scavenging effects, which may be correlated with the presence of their chemical constituents.

### 2.2. Non-Toxic Concentration and Photo-Protective Effects of the Extract on UVB-Irradiated Keratinocyte Cells

**Figure 2:**
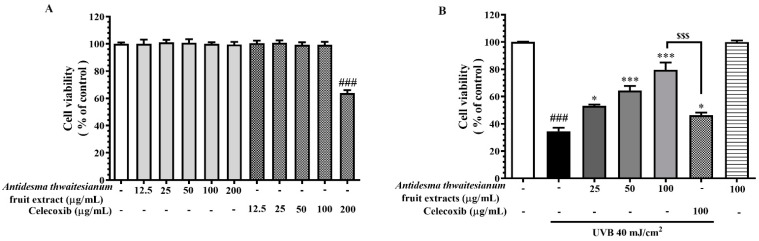
The photoprotective effect of the fruit extract on the keratinocyte cells. (**A**) shows cell viability following treatment with various concentrations of the fruit extract or celecoxib control for 24 h. (**B**) shows cell viability after pre-incubating with the fruit extract for 2 h, exposing a single UVB irradiation and further incubating for 24 h. The results are represented as mean ± SEM (n = 6) and analyzed with Tukey’s test. ^###^ *p* < 0.001 versus the control group; * *p* < 0.05 and *** *p* < 0.001 versus UVB-irradiated cells alone. The statistical difference between the extract and celecoxib at 100 µg/mL was at ^$$$^ *p* < 0.001.

To identify the protective effect against UVB-induced cytotoxicity, we firstly determined a safety dose used in this study. We measured cell viability following the treatment with fruit extracts at various concentrations, 0–200 µg/mL for 24 h as well as the positive control celecoxib in the same range. Celecoxib is a commercial anti-inflammatory drug that is prescribed for skin inflammation. We found that the fruit extract had negligible cytotoxicity at all dosages, while celecoxib exhibited cytotoxicity at 200 µg/mL (Figure 2A). We, therefore, experimented with the highest dose of 100 µg/mL (Figure 2B). We found that UVB induced cytotoxicity and lowered cell viability to 34.56 ± 2.58%. The pre-treatment with the fruit extract recovered cell viability to 53.18 ± 0.89, 64.44 ± 3.35, and 79.65 ± 5.26% following 25, 50, and 100 µg/mL, respectively. All treated doses exhibited significant cell recovery compared to the untreated cells. In addition, the pre-treatment with the fruit extract exhibited a greater cell survival compared to celecoxib, about 35% at the same dose of 100 µg/mL. This result suggests the potential effect of the fruit extract at 100 µg/mL, effectively protecting cells from UVB-induced cytotoxicity.

### 2.3. The Antioxidant and Anti-Apoptotic Effects of the Extract on UVB-Irradiated Keratinocyte Cells

**Figure 3:**
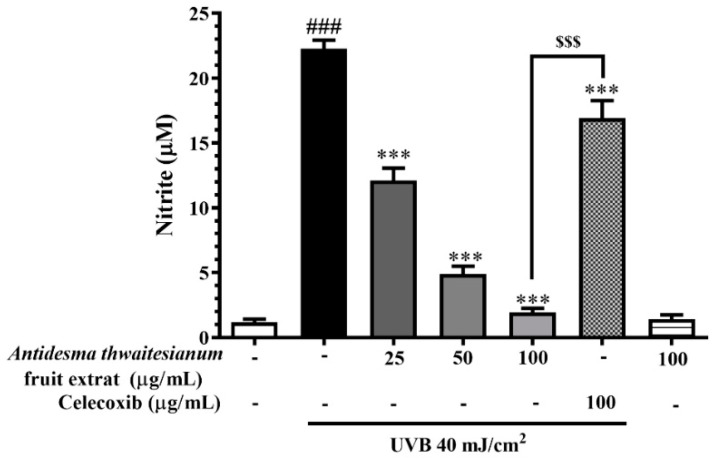
The NO free-radical scavenging effect of the fruit extract on UVB-irradiated keratinocyte cells. The cells were pre-incubated with various concentrations of the fruit extract before exposure to single UVB irradiation, followed by additional cell incubation for 24 h. The results are represented as mean ± SEM (n = 8) and analyzed with Tukey’s test. ^###^ *p* < 0.001 versus the control group, *** *p* < 0.001 versus UVB-irradiated cells alone. The statistical difference between the extract and celecoxib at 100 µg/mL was at ^$$$^ *p* < 0.001.

The UVB irradiation increased the intracellular NO in keratinocytes. The UVB exposure at 40 mJ/cm^2^ rose the nitrite concentration to 22.27 ± 0.65 µM. We showed that the treatment with the fruit extract at all the tested dosages did not induce NO generation compared with the non-irradiation conditions. The pre-treatment with the fruit extract 2 h before UVB exposure resulted in a decrease in NO generation, which subsequently diminished nitrite levels, a stable metabolite of NO. Following UVB irradiation, the fruit extract at 25, 50, and 100 µg/mL significant diminished the nitrite concentration to 12.11 ± 0.95, 4.89 ± 0.59, and 1.92 ± 0.31 µM, respectively. Celecoxib at 100 µg/mL had a moderate NO inhibitory effect, while the fruit extract at the lowest dose of 25 µg/mL could prevent NO production in a greater level than the positive control, and this inhibitory effect was displayed in a dose-dependent manner. This result indicates that the fruit extract did not induce intracellular NO, which plays an important role in inflammation. In addition, we showed that the fruit extract had a protective property against UVB-induced NO production in keratinocytes. Together, these findings indicate the anti-inflammatory effect of the fruit extract through the prevention of NO production (Figure 3).

**Figure 4:**
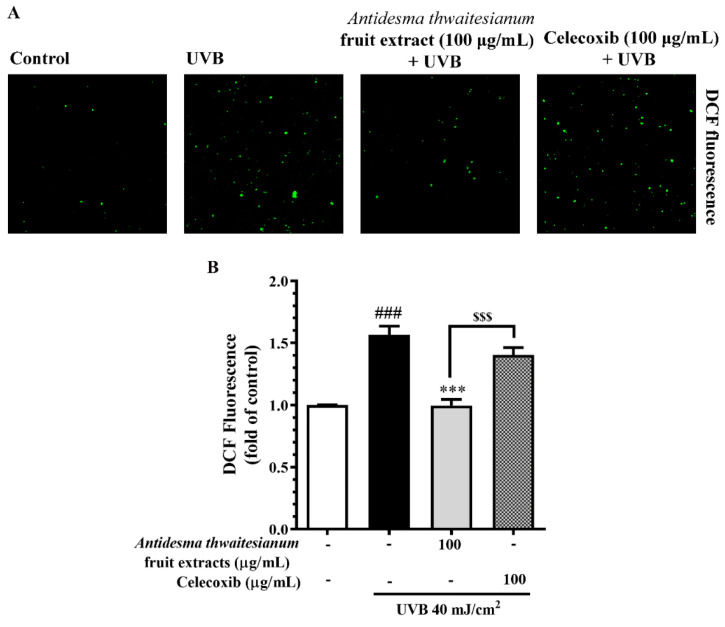
Intracellular ROS production was determined by H_2_DCFDA staining assay. The ROS scavenging effect of the fruit extract was monitored by the reduction in fluorescence. (**A**) shows the DCF fluorescent staining after pre-incubating with the fruit extract for 2 h, exposed to single UVB irradiation, and further incubating for 45 min. (**B**) shows the relative fluorescence intensities, which are presented as mean ± SEM (n = 6) and analyzed with Tukey’s test. ^###^ *p* < 0.001 versus the control group, *** *p* < 0.001 versus UVB-irradiated cells alone. The statistical difference between the extract and celecoxib at 100 µg/mL was at ^$$$^ *p* < 0.001.

**Figure 5:**
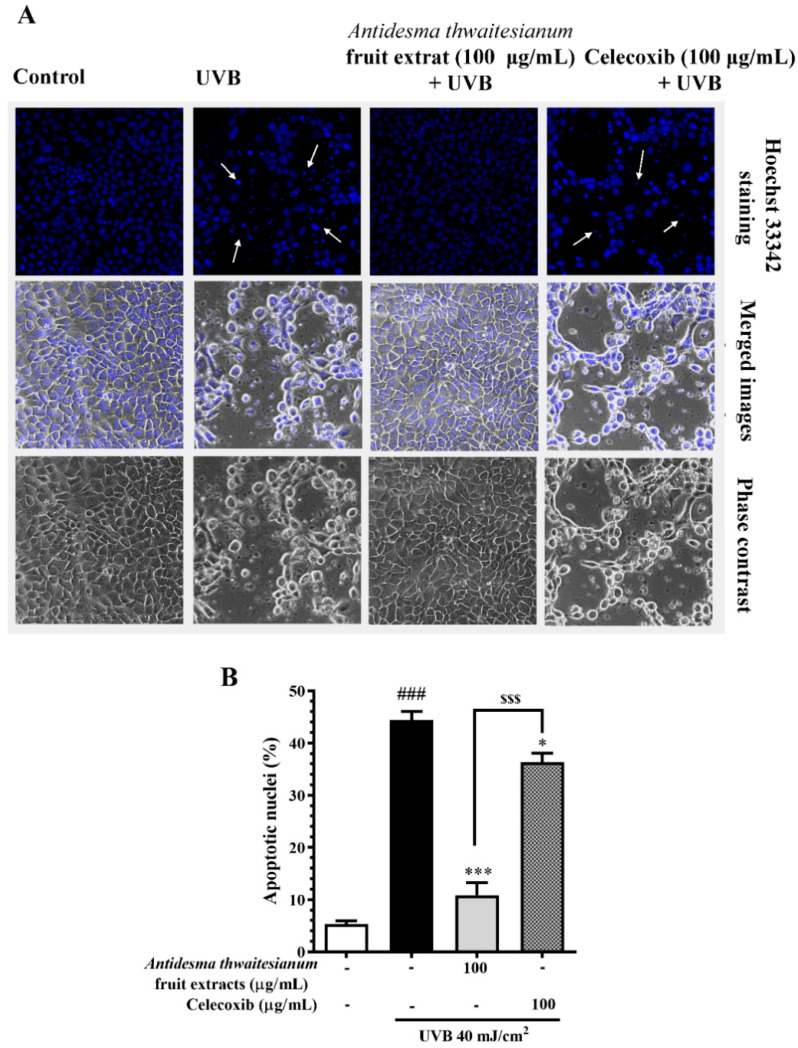
The photoprotective effect of the fruit extract following UVB-induced apoptosis. (**A**) shows the Hoechst 33342 nuclei staining after pre-incubating cells with the fruit extract for 2 h, exposed to single UVB irradiation, and further incubating for 24 h. (**B**) shows the relative fluorescence intensities, which are represented as mean ± SEM (n = 6) and analyzed with Tukey’s test. The inserted image shows the magnified apoptotic nuclei of the UVB-treated cells (40× magnification of objective lens). ^###^ *p* < 0.001 versus the control group, * *p* < 0.05 and *** *p* < 0.001 versus UVB-irradiated cells alone. The statistical difference between the extract and celecoxib at 100 µg/mL was at ^$$$^ *p* < 0.001.

In addition, we further determined the ROS scavenging capacity of the extract and celecoxib by using the ROS assay (Figure 4). Upon single UVB exposure, the cells increased the intracellular ROS to about 1.56 ± 0.12-fold that of the non-irradiated conditions (control). We found that the pre-treatment with the extract at 100 µg/mL reduced the intracellular ROS to about 0.99 ± 0.05 that of the control, while celecoxib at the same dose had less a potent effect. Celecoxib treatment significantly lowered the intracellular ROS to 1.40 ± 0.59-fold that of the control. This indicates that the fruit extract contained antioxidant properties, while the anti-inflammatory drug celecoxib had a small effect on preventing ROS production. In addition to NO and ROS production, we showed that a single UVB exposure of 40 mJ/cm^2^ led to the apoptosis of more than 40% (Figure 5). The pre-incubation with celecoxib at 100 µg/mL alleviated UVB-induced cell death, while the fruit extract exhibited a greater potential than celecoxib at the same concentration. We showed that UVB exposure decreased cell viability. The majority of cell death was related to nuclear chromatin condensation and DNA fragmentation, after which cells ultimately undergo apoptosis. Along with DNA damage, we found an increase in intracellular NO and ROS, which was a stress response to prevent further damage. However, the accumulation of NO and ROS was also harmful to the cells. In this study, we showed that the fruit extract reduced the intracellular NO and ROS following UVB-induced cytotoxicity. The reduction in NO and ROS with the fruit extract treatment correlated with the recovery of cell survival and alleviated UVB-induced apoptosis. In addition, the fruit extract protected cells from UVB-induced apoptosis in a greater manner than celecoxib of about 25%, which may be the case by the synergistic effect of the multiple antioxidants in the fruit extract.

### 2.4. The Effects of the Extract on UVB-Induced Cellular p38 and JNK Phosphorylation

**Figure 6:**
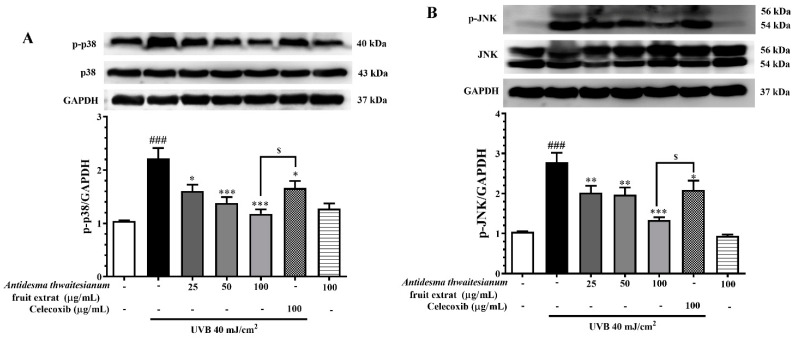
The dose-dependent effects of the fruit extract on p38 and JNK phosphorylation. Cells were pre-incubated with the fruit extract for 2 h before exposure to single UVB irradiation and further incubated for (**A**) 6 h and (**B**) 1 h. The proteins of interest were determined by Western blot analysis. The results are presented as mean ± SEM (n = 8) and analyzed with Tukey’s test. ^###^ *p* < 0.001 versus the control group; * *p* < 0.05, ** *p* < 0.01, and *** *p* < 0.001 versus UVB-irradiated cells alone. The statistical difference between the extract and celecoxib at 100 µg/mL was at ^$^ *p* < 0.05.

UVB has been previously described as a stress activator of two MAPK signaling pathways: p38 and JNK [13,14]. The activation of MAPK occurs through the phosphorylation of their tyrosine residue. In this study, the level of phospho-p38 increased 2.21 ± 0.19-fold after UVB exposure, while p38 protein level was similar in all conditions. The pre-treatment with the fruit extract could attenuate the UVB-induced MAPK activation in a dose-dependent manner. We showed that the pre-treated cells with the extract 25, 50, and 100 µg/mL significantly decreased the phospho-p38 to 1.6 ± 0.11, 1.38 ± 0.11, 1.18 ± 0.18, respectively (Figure 6A). The reduction in phospho-p38 mediated by the fruit extract was better than celecoxib. In addition, we showed that the level of phospho-JNK was negligible under non-irradiation conditions, while UVB exposure increased phospho-JNK to 2.78 ± 0.23 but not JNK protein level. The UVB-induced JNK activation was also diminished following the pre-treatment with the fruit extract. We found that phospho-JNK reduced to 2.0 ± 0.17, 1.97 ± 0.18, 1.33 ± 0.07 following pre-treatment with 25, 50, and 100 µg/mL fruit extract, respectively (Figure 6B). The reduction in phospho-p38 and phospho-JNK by the fruit extract was exhibited in a dose-dependent manner and was more potent than celecoxib at the same dose (*p*-value < 0.05).

### 2.5. The Effects of the Extract on UVB-Induced COX-2 and PGE_2_ Production

**Figure 7:**
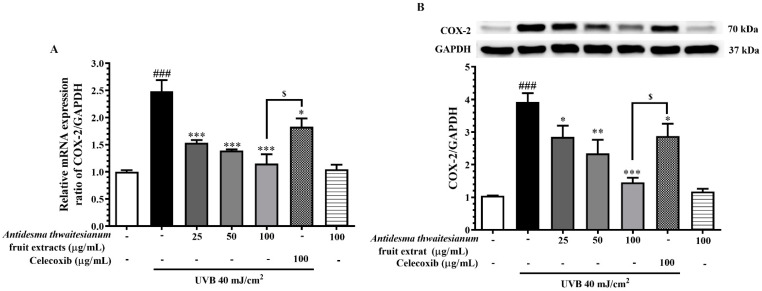
The alterations of (**A**) mRNA and (**B**) protein expression levels of COX-2 upon treatment with various concentrations of the fruit extract. Cells were pre-incubated with the fruit extract before exposure to single UVB irradiation and further incubated for 24 h. The mRNA was determined by real-time PCR and the proteins were determined by Western blot analysis. The results are presented as mean ± SEM (n = 6) and analyzed with Tukey’s test. ^###^ *p* < 0.001 versus the control group; * *p* < 0.05, ** *p* < 0.01, and *** *p* < 0.001 versus UVB-irradiated cells alone. The statistical difference between the extract and celecoxib at 100 µg/mL was ^$^ *p* < 0.05.

The activation of the MAPK signaling pathway resulted in several cellular responses, such as apoptosis and inflammation. Several previous studies revealed that MAPK activates gene expression through the translocation of transcription factors. One of the gene inductions in responding to UVB exposure was *COX-2*, which in turn stimulates the pro-inflammatory response [29,30] and subsequently generates skin tumorigenesis [31,32]. In this study, we found that UVB induced the expression level of *COX-2* mRNA to 2.48 ± 0.20-fold that of the non-irradiation control, as well as increased COX-2 protein level to 3.92 ± 0.27-fold that of the control. We showed that the pre-treatment with the fruit extract at 25, 50, and 100 µg/mL significantly downregulated *COX-2* mRNA expression to 1.54 ± 0.05, 1.39 ± 0.02, 1.15 ± 0.17, respectively (Figure 7A). Additionally, COX-2 protein levels corresponded to mRNA levels, which were 2.85 ± 0.34, 2.34 ± 0.41, and 1.46 ± 0.14 following the pre-treatment with the fruit extract at 25, 50, and 100 µg/mL, respectively (Figure 7B). This result indicates that the fruit extract modulated the COX-2 protein level through the downregulation of the gene expression, and the repression of the gene occurred in a dose-dependent manner. We found that celecoxib at 100 µg/mL also reduced the *COX-2* mRNA and protein levels, but was less potent than the fruit extract. This result also correlated with the attenuation of its upstream MAPK signaling pathway.

**Figure 8:**
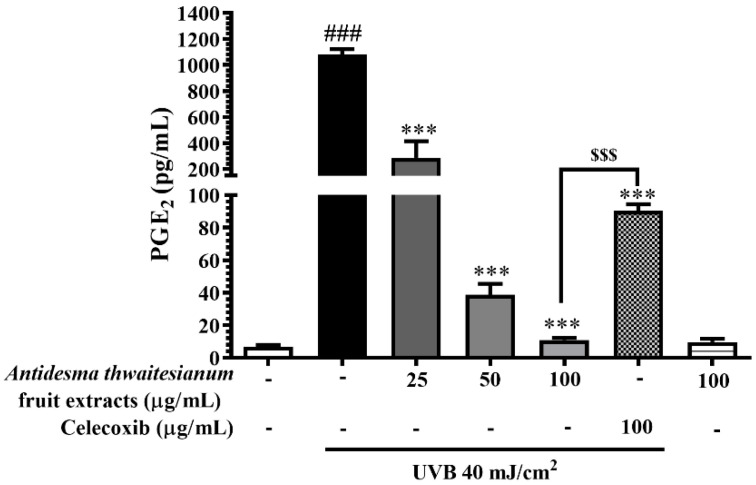
The dose-dependent effects the fruit extract on PGE_2_ level. Cells were pre-incubated with the fruit extract for 2 h before exposure to single UVB irradiation and further incubated for 24 h. The cell culture media were used to determine the PGE_2_ level by ELISA assay. The results are represented as mean ± SEM (n = 6) and analyzed with Tukey’s test. ^###^ *p* < 0.001 versus the control group, *** *p* < 0.001 versus UVB-irradiated cells alone. The statistical difference between the extract and celecoxib at 100 µg/mL was ^$$$^ *p* < 0.001.

The induction of the COX-2 enzyme increased its target PGE_2_ catabolism. PGE_2_ is the pro-inflammatory mediator that stimulates skin redness, sunburn, and inflammation. In this study, UVB-irradiated cells increased the production of PGE_2_ to 1,082 ± 40.40 pg/mL, while the pre-treatment with the fruit extract lowered PGE_2_ production. We showed that the level of PGE_2_ was associated with the level of COX-2 enzyme. The reduction in PGE_2_ levels corresponded with the decrease in COX-2 levels, which were 286.3 ± 129.1, 38.64 ± 6.7, and 10.92 ± 1.35 pg/mL upon treatment with 25, 50, and 100 µg/mL extract, respectively (Figure 8). We conclude that the fruit extract mediated UVB-irradiated protection through the inhibition of intracellular NO and ROS and subsequently attenuated MAPK-activated apoptosis and pro-inflammation.

## 3. Discussion and Conclusions

In the past few years, the discovery of new herbal medicines harboring UV-protection properties has gained great interest in the skincare industry. In this study, we utilized the HaCaT cell line to evaluate the photo-protective effects of the *A. thwaitesianum* fruit extract upon single UVB exposure. HaCaT is a keratinocyte cell line that is widely used as a cell-based model for studying cellular responses to UV and molecular mechanisms underlying anti-inflammatory activities [13,33]. UVB refers to radiation at the wavelength of 280–320 nm. It is an extrinsic environmental stimulus that can penetrate deeply through the epidermis layer of the skin. UVB exposure induces the generation of intracellular ROS and is mainly attributed to skin inflammation, such as erythema and edema [34]. In this study, we demonstrated the antioxidant and nitric oxide scavenging properties of this fruit extract. In addition, the molecular analysis revealed the decrease in MAPK-activated stress response, including cell death and inflammation, following fruit extract pre-treatment.

In correlation with previous studies, we found that a UVB exposure of 40 mJ/cm^2^ had a significant reduction in cell viability and increment in NO and ROS production [11,12,14]. However, the pre-incubation with the ethanol extract of *A. thwaitesianum* fruit (25, 50, and 100 μg/mL) before UVB irradiation increased the percentage of cell viability in a concentration-dependent manner (Figure 2B). In addition, the *A. thwaitesianum* fruit extract at all tested dosages had minimal cytotoxicity, which indicates the low toxicity of this fruit extract. We believe that the protective effect of the *A. thwaitesianum* fruit extract may be related to its chemical components, polyphenols, and flavonoids. We identified the presence of ferulic acid, caffeic acid, vanillic acid, protocatechuic acid, and cyanidin, which have been previously described as antioxidant compounds with anti-inflammatory and photo-protective properties [35,36,37,38,39]. The DPPH assay revealed that the fruit extract could act as electron and proton donors that stabilize DPPH free radicals. In addition, we found that the increase in intracellular ROS and NO following UVB exposure was eliminated by the pre-treatment with the *A. thwaitesianum* fruit extract. Additionally, we found that the fruit extract attenuated apoptosis and recovered cell viability (Figure 2B and Figure 5).

Following UVB exposure, we monitored the MAPK signaling pathway, p38, and JNK, in response to stress activation. The phosphorylation of MAPK via their canonical upstream kinase activates the downstream response through the induction of gene expression and cellular response. The activation of MAPK in response to UVB exposure resulted in DNA damage and cell death. We showed that the single exposure of UVB increased the phosphorylated-p38 and -JNK but did not affect p38 and JNK protein levels. Thus, the reduction in the phosphorylated form could attenuate stress-induced MAPK responses. We showed that the inhibition of the MAPK pathway by the *A. thwaitesianum* fruit extract occurred at the upstream level, and thus resulted in cell recovery (Figure 6A,B). Despite the aforementioned cell survival, we demonstrated the reduction in the *COX-2* gene and protein expression levels (Figure 7A,B). COX-2 is the enzyme that is expressed in response to prostanoid biosynthesis in inflammation, particularly PGE_2_. Following UVB exposure, previous studies revealed the upregulation of COX-2 and PGE_2_ involving skin cancer [15,40]. In this study, we showed that the pre-treatment with the *A. thwaitesianum* fruit extract reduced COX-2 as well as lowering the synthesis of the pro-inflammatory mediator PGE_2_ (Figure 8). This result indicates the anti-inflammatory effect of the *A. thwaitesianum* fruit extract, which can be an effective adjuvant strategy for preventing skin inflammation and cancer after chronic exposure to UVB radiation. Our results are correlated with previous studies showing anti-inflammatory activity and free-radical scavenging activity [24,25,41].

Celecoxib has been described as a COX-2 inhibitor that is prescribed to treat skin inflammation. It has been reported to reduce the risk of skin tumorigenesis and contain anti-inflammation properties upon chronic UVB exposure [42,43]. In this study, we used celecoxib as a positive control. We demonstrated that the *A. thwaitesianum* fruit extract exhibited a greater potential than celecoxib at the same dose. This may be due to the presence several antioxidants, which exerted their synergistic effect [35,36,37,38,39].

Taken together, the molecular mechanisms underlying UVB-triggered keratinocyte cytotoxicity and inflammatory response were initiated by excess intracellular ROS and NO generation, which subsequently induced oxidative stress. The stress activation of p38 and JNK signaling pathways, in turn, stimulated cellular responses, such as cell death and inflammation. The pre-treatment with the *A. thwaitesianum* fruit-extract-enriched polyphenolic and flavonoid contents could alleviate cellular stress response, which results in the cell survival recovery and the decrease in the inflammatory mediator.

In conclusion, the present study firstly demonstrated the potential photo-protective and anti-inflammation effects of the ethanol extract of the *A. thwaitesianum* fruit against UVB-activated keratinocyte toxicity. Its effects were directly involved in the antioxidant properties of polyphenolic contents. Our finding offers a new herbal medicine as an alternative ingredient for skincare products to alleviate UVB-induced skin inflammation. However, the safety usage and the application of this fruit extract should be further investigated in animal models.

## 4. Materials and Methods

### 4.1. Plant Preparation and Extraction

*A. thwaitesianum* fruits were obtained from Lam Takhong Research Station, Thailand Institute of Scientific and Technological Research, Thailand. The oval shape of the ripe red fruits was blended using an electronic blender. Then, the sample was lyophilized in a freeze dryer. A total of 50 g of sample was immersed in 500 mL of 95% ethanol for 3 days at room temperature and percolated through Whatman No. 1 filter paper to remove residues. The residues were repeatedly immersed in the same volume of ethanol three times and followed by filtration. The total filtrate was evaporated and concentrated to dried powder by using a vacuum rotary evaporator. The powder was kept at −20 °C in a sterile tube or dissolved in dimethyl sulfoxide (DMSO) to 200 mg/mL stocking solution. The final concentration of DMSO in all tested experiment was 0.1% in every concentration.

### 4.2. Determination of Phenolic and Flavonoid Contents

As described in previous studies, the total phenolic content was determined by the Folin–Ciocalteu colorimetric method, while the total flavonoid content was determined by aluminum chloride colorimetric methods [44,45]. In brief, a mixture of 10 μL of the extract in 790 μL deionized water was mixed with 50 μL Folin–Ciocalteu’s phenol reagent. The mixture was incubated at room temperature for 5 min before being added with 150 μL saturated Na_2_CO_3_ solution and incubated at room temperature for 50 min. The total polyphenol contents were determined by spectrophotometry at 765 nm. The data were expressed as milligram gallic acid equivalents (GAE) per gram of extract. For the determination of flavonoid content, 100 μL of the extract was dissolved in 560 μL deionized water and then mixed with 20 μL potassium acetate and 300 μL of 95% ethanol. A total of 20 μL of 10% aluminum chloride was added to the mixture and incubated at room temperature for 30 min. The flavonoid contents were determined by spectrophotometry at 415 nm and showed as quercetin equivalents (QE) per gram of extract.

### 4.3. Determination of In Vitro DPPH Scavenging Activity

This assay aimed to evaluate the DPPH scavenging capability of the extract in vitro. As mentioned in our previous work [46], the reduction in DPPH radical absorbance was monitored using a microplate reader (Bio-Tek Instruments, Winooski, VT, USA) at 515 nm. The percentage inhibitions of DPPH radical scavenging activity were calculated using the following formula.

DPPH radical scavenging % = [(A0 − A1)/A0] × 100

A0 is the absorbance of the DPPH solution.

A1 is the absorbance of the sample.

### 4.4. High-Performance Liquid Chromatography (HPLC) Analysis of the Extract

HPLC analysis was performed to identify the bioactive compounds in the extract. The method was previously described with some modifications [47]. In brief, the extract was dissolved with deionized water to obtain four different concentrations and subjected to HPLC analysis (Waters 2695 HPLC System with 2487 UV-VIS Detector, Marshall Scientific, Hampton, NH, USA). The sample mixture was diluted with 0.5% of acetonitrile and trifluoroacetic acid solution as a mobile phase. The retention time peaks were analyzed using a gradient system with a flow of 1 mL/min. The signal was detected using a UV-VIS detector with a wavelength of 280 nm and a run time of 60 min.

### 4.5. Cell Culture and UVB Treatment

HaCaT cells, an immortalized human keratinocyte line, was purchased from Cell Lines Service (Eppelheim, Baden-Württemberg, Germany) and used to evaluate the anti-inflammatory mechanism of the extract in vitro [33]. Cells were cultured in high glucose Dulbecco’s modified Eagle medium (DMEM) (GIBCO, Carlsbad, CA, USA), with 10% heat-inactivated fetal bovine serum (HiMedia, Mumbai, India), and maintained in the CO_2_ incubator at 37 °C, 5% CO_2_ air atmosphere, and 95% humidity. The attached cells were rinsed with phosphate-buffered saline (PBS) before being irradiated with UVB irradiation at 40 mJ/cm^2^ (BIO-LINK^®^, Vilber Lourmat UV-Crosslinker, Deutschland GmbH, Eberhardzell, Germany). The cells were immediately added to a cell culture medium and placed in the 37 °C, CO_2_ incubator before performing further experiments [12].

### 4.6. Cell Viability Determination by Resazurin Assay

The cells were seeded at a density of 4 × 10^4^ cells/well in 96-well plates and allowed to attach overnight. To determine the sublethal dose, different concentrations of the extract or celecoxib were added to each well and incubated at 37 °C, 5% CO_2_ incubator for 24 h. To assess the photoprotective effect, the cells were pre-incubated with the extract or celecoxib for 2 h before exposing a single UVB irradiation. Cells were then incubated in 37 °C, CO_2_ incubator for 24 h before measuring cell viability using Resazurin assay. In brief, 20 µL resazurin solution was directly added to each well (Resazurin assay kit; Sigma-Aldrich, St. Louis, MO, USA). After incubation for 3 h, the fluorescent intensities were measured at emission 530/excitation 590 nm using a microplate reader (Bio-Tek Instrument, Winooski, VT, USA). The cell viability was calculated as a percentage compared to untreated cells.

### 4.7. Nitric Oxide (NO) Scavenging Activity by Griess Assay

As previously described, the NO production produced was investigated by measuring the accumulated nitrite in the culture media [12]. The cells were plated onto a 6-well plate at a seeding density of 2.5 × 10^6^ cells/well and pre-incubated with the extracts for 2 h before being exposed to UVB. After the exposure, cells were then incubated in 37 °C, 5% CO_2_ incubator for 24 h. A total of 100 μL of the culture media of each condition was mixed with 100 μL of Griess reagent in a 96-well plate and incubated at room temperature for 10 min (NO assay kit; Sigma-Aldrich, St Saint Louis, MO, USA). The absorbance of the reaction mixtures was measured at 540 nm using a microplate reader (Bio-Tek Instrument, Winooski, VT, USA).

### 4.8. Antioxidant Activity by ROS Assay

The 2’,7’-dichlorodihydrofluorescein diacetate (H_2_DCFDA) assay kit (Sigma-Aldrich, St Saint Louis, MO, USA) was used to evaluate intracellular reactive oxygen species upon UVB exposure [48]. The purpose of this experiment was to compare the antioxidant effects of the extract and celecoxib. Cells were seeded into an 8-chamber cover glass and treated with 20 µM of H_2_DCFDA for 30 min in the dark. The cells were then washed with PBS and pretreated with the extract or celecoxib for 2 h before UVB exposure and incubated for a further 45 min. The fluorescent images were promptly taken using the confocal fluorescent microscope (Olympus, Japan). The ROS fluorescent intensity was calculated from the data average of three different areas by using Olympus software.

### 4.9. Determination of Interested Proteins by Western Blot Analysis

Cells were pre-incubated with the extract at 25, 50, and 100 µL for 2 h before single exposure to UVB radiation and further incubation at 37 °C, 5% CO_2_ incubator for optimal time points. Following treatment, the cells were lysed in RIPA buffer containing with 1% protease inhibitor cocktail and phosphatase inhibitor cocktail. The lysates were centrifuged at 12,000 rpm at 4 °C for 15 min. The supernatants were then collected for the determination of protein amount using a Bradford assay kit (Cell Signaling Technology, Danvers, MA, USA). Equal amounts of total protein (60 μg) were boiled for 5 min and ran on a 12.5% SDS-polyacrylamide gel. The protein on a gel was then transferred to nitrocellulose membranes and blocked in 5% nonfat dry milk in TBS-T buffer (Tris-buffer saline containing 0.1% tween 20) for 1 h at room temperature on the shaker. After being blocked, the Western blot membranes were subsequently incubated with primary antibodies against p-p38 (Cell Signaling Technology, Danvers, MA, USA), p-JNK, and COX-2 (Abcam, Cambridge, MA, USA). The anti-GADPH monoclonal antibody was used as an internal control of the interested proteins. The Western membranes were further incubated with secondary antibodies at 4 °C overnight on the shaker. The protein bands were developed using an Immobilon Forte Western HRP substrate (Merck KGaA, Darmstadt, Germany) and imaged using gel documentation: Alliance Q9 Advanced Chemiluminescence Imager (UVITEC, Cambridge CB4 OWS England, and United Kingdom). The relative protein intensities were analyzed using the ImageJ program and calculated as a fold of control.

### 4.10. Determination of PGE_2_ Level by ELISA Assay

Cells were pre-treated with extract at various concentrations for 2 h and then exposed to UVB. The ELISA technique was carried out to evaluate the PGE_2_ level in cell culture media following the manufacturer’s recommendations (Abcam, Cambridge, MA, USA). In brief, the cell culture media were added to the ELISA plate and incubated at room temperature. The level of PGE_2_ was determined by colorimetric absorbance at 405 nm using a microplate reader (Bio-Tek Instrument, Winooski, VT, USA).

### 4.11. Determination of Gene Expression by Real-Time PCR

RNA was extracted by using an RNA extraction kit (Favorgen, Ping-Tung, Taiwan). The total amount of RNA (2 μg) was used to synthesize cDNA by using iScript^TM^ reverse transcription Supermix (Bio-Rad, Hercules, CA, USA). The synthesized cDNA was then amplified by SentiFast SYBR^®^ Hi-ROX mix (Bioline, TN, USA), according to the manufacturer’s recommendations. The relative quantification of gene expression was analyzed with StepOnePlus (Applied biosystem, Waltham, MA, USA). The relative gene expression was calculated based on the reference gene; GADPH using the delta delta Ct’ values. List of primers used in the experiment was showed in Table 3.

### 4.12. Statistical Analysis

The four to six biological replicates were analyzed with one-way ANOVA analysis followed by Tukey’s test using GraphPad Prism program version 8 (GraphPad software, San Diego, California, USA). All data were expressed as mean ± SEM. The given *p*-values of less than 0.05 was considered statistically significant.

## Figures and Tables

**Table 1:** The active compounds contained in the *A. thwaitesianum* fruit extract quantified by HPLC analysis.

Compounds	*A. thwaitesianum* Fruit Extract (μg/mL)
Ferulic acid	798.13
Caffeic acid plus Vanillic acid	510.79
Protocatechuic	487.76
Cyanidin	52.70

**Table 2:** IC_50_ DPPH and total phenolic and flavonoid contents of the *A. thwaitesianum* fruit extract.

*A. thwaitesianum* Fruit Extract	Phenolic Content (mg Gallic Acid Equivalent/g Crude Extract)	Flavonoid Content (mg Quercetin Equivalent/g Crude Extract)	IC_50_ of DPPH (mg/mL)
EtOH extract	29.115 ± 0.528	1.237 ± 0.104	10.94
Trolox	-	-	0.17

**Table 3:** List of primers used in this study.

Genes	Forward Primer	Reverse Primer
*COX-2*	TGAGCATCTACGGTTTGCTG	TGCTTGTCTGGAACAACTGC
*GAPDH*	TGAGCATCTACGGTTTGCTG	TGCTTGTCTGGAACAACTGC

## Data Availability

Not applicable.
